# Allorecognition Unveiled: Integrating Recent Breakthroughs Into the Current Paradigm

**DOI:** 10.3389/ti.2024.13523

**Published:** 2024-11-11

**Authors:** Xavier Charmetant, Gavin J. Pettigrew, Olivier Thaunat

**Affiliations:** ^1^ Centre International de Recherche en Infectiologie, INSERM U1111, Université Claude Bernard Lyon I, CNRS UMR5308, Ecole Normale Supérieure de Lyon, University Lyon, Lyon, France; ^2^ Department of Transplantation, Nephrology and Clinical Immunology, Edouard Herriot Hospital, Hospices Civils de Lyon, Lyon, France; ^3^ Lyon-Est Faculty of Medicine, Claude Bernard University (Lyon 1), Villeurbanne, France; ^4^ Department of Surgery, University of Cambridge, Cambridge, United Kingdom

**Keywords:** adaptive immunity, innate immunity, allorecognition pathways, graft rejection, pathophysiology

## Abstract

In transplantation, genetic differences between donor and recipient trigger immune responses that cause graft rejection. Allorecognition, the process by which the immune system discriminates allogeneic grafts, targets major histocompatibility complex (MHC) and minor histocompatibility antigens. Historically, it was believed that allorecognition was solely mediated by the recipient’s adaptive immune system recognizing donor-specific alloantigens. However, recent research has shown significant roles for innate immune components, such as lymphoid and myeloid cells, which are sometimes triggered by the mere absence of a self-protein in the graft. This review integrates recent breakthroughs into the current allorecognition paradigm based on the well-established direct and indirect pathways, emphasizing the semi-direct pathway where recipient antigen-presenting cells (APCs) acquire donor MHC molecules, and the inverted direct pathway where donor CD4^+^ T cells within the graft activate recipient B cells to produce donor-specific antibodies (DSAs). The review also explores the role of natural killer (NK) cells in both promoting and inhibiting graft rejection, highlighting their dual role in innate allorecognition. Additionally, it discusses the emerging understanding of myeloid cell-mediated allorecognition and its implications for initiating adaptive immune responses. These insights aim to provide a more comprehensive understanding of allorecognition, potentially leading to improved transplant outcomes.

## Introduction

The current dogma in transplant immunology defends that the genetic differences between the donor and recipient play a crucial role in shaping the outcomes of organ and tissue transplants. These differences lead to the expression of alloantigens, which serve as markers for the immune system to distinguish between self and non-self-tissues.

Alloantigens are categorized into two main types: major histocompatibility complex (MHC) and minor histocompatibility glycoprotein antigens. The latter include all the molecules other than the MHC which are polymorphic and therefore differ between the donor and the recipient. These two categories of alloantigens differ in size—six molecules of MHC (three of class I and three of class II) versus a myriad of minor histocompatibility antigens—and level of polymorphism (i.e., the variability of the amino-acid composition, which is very high for MHC antigens and more limited for minor histocompatibility antigens) [1]. Furthermore, these two categories of alloantigens are recognized by the recipient’s immune system through distinct mechanisms (grouped under the umbrella term allorecognition). The complex immune responses that are triggered lead to the various types of graft rejections commonly diagnosed in transplant patients.

Despite extensive studies over the past four decades, allorecognition remains a critical area of interest for transplant physicians. These professionals frequently encounter clinical situations that are not adequately explained by the current conceptual framework of transplant immunology. For instance, some episodes of cellular rejection occur late after transplantation, long after the disappearance of donor-derived passenger antigen-presenting cells. In contrast, very early flares of donor-specific antibodies (frequently observed after lung or intestinal transplantation) arise too quickly to result from a classical humoral response. Finally, while it is still largely believed that allorecognition is solely the domain of the recipient’s adaptive immune system, recent publications have revealed a significant role for both the lymphoid and myeloid components of the innate immune system. Furthermore, the latter sometimes discriminate the graft not because it expresses donor-specific alloantigens, but because of the lack of expression of self-proteins.

In this review, we aim to provide a concise overview of the established theories that form the basis of the current understanding of allorecognition. Building on this foundation, we incorporate recent immunological discoveries that address existing theoretical gaps, with the goal of offering a more comprehensive understanding of allorecognition.

## A Brief History of Allorecognition

George Snell’s groundbreaking work in the mid-20th century led to the discovery that graft rejection was primarily driven by genetic differences in the MHC.

The discovery that intact donor-specific MHC molecules on the surface of passenger APCs could be “directly” recognised by a large proportion of recipient’s T cell clones introduced the concept of direct allorecognition, which was considered the initial step of the pathophysiological cascade of T-cell mediated rejection (TCMR). However, this model did not fully explain certain clinical observations, such as late TCMR episodes occurring long after the disappearance of donor-derived passenger APCs. In 2004, Herrera et al. proposed the concept of semi-direct allorecognition, in which the recipient’s APCs present intact, unprocessed donor MHC molecules on their surface, following the capture of extracellular vesicles. Although this was a significant advance, it took considerable time to fully elucidate the details of this pathway, up to the description of the “three-cell cluster” model, where a single recipient APC presents both intact donor MHC and processed peptides, facilitating interactions between CD4^+^ and CD8^+^ T cells. This mechanistic understanding was critical in refining our view of how T cell-mediated alloimmune responses are sustained over time following transplantation. In fact some works suggest that even early episode of TCMR rely rather on the semi-direct pathway than the direct pathway [2].

Meanwhile, in the 2000s, Paul Terasaki proposed the humoral theory of organ rejection, in which the generation of donor-specific antibodies results on an antigenic recognition mechanism described years earlier by Lanzavecchia (1985), and which has come to be known as “indirect allorecognition” in the field of transplantation. This model helped explain antibody-mediated damages to graft endothelium and their role in long-term outcomes.

Parallel to these developments, the concept of “missing-self” was introduced in 1986, whereby NK cells can detect and destroy cells that lack self-MHC molecules. However, it was not until 2019 that this principle was fully applied to the field of transplantation, where NK cells were found to mediate allorecognition and directly contribute to graft rejection, particularly in the context of chronic microvascular damage.

Most recently, in the late 2010s, the concept of innate myeloid allorecognition has emerged. Fadi Lakkis’s group has demonstrated that monocytes can directly recognize allogeneic non-self in a MHC-independent manner. This discovery adds another layer of complexity to allorecognition. However, whether this mechanism functions in clinical transplantation remains to be proven, highlighting the long path from fundamental immunological discoveries to their practical application in transplantation.

## Allorecognition Pathways Leading to T Cell-Mediated Rejection

MHC molecules allow certain immune cells (such as dendritic cells and B cells) to present antigens (in the form of peptides) to T cells. They are made of a framework region and a binding groove. The amino acids that make up the latter define the peptide to which it will bind with the greatest affinity. The T-cell receptor (TCR) interacts with a complex zone, comprising both framework parts of the MHC and the antigen itself.

In a given individual, the process of thymic education of T lymphocytes shapes TCR repertoire. The clones whose TCR do not recognise self MHC are eliminated (“positive selection”), while self-reactive clones are either eliminated (“negative selection”) or selected to differentiate into natural T reg [3]. In this manner, the TCR specificity of peripheral T cells is theoretically limited to recognizing self-MHC molecules presenting non-self-peptides, a concept for which Doherty and Zinkernagel were awarded the Nobel Prize [4]. This immunological dogma however does not explain why transplanted organs are targeted with such intensity by the recipient’s cytotoxic T cells. The occurrence of T cell-mediated rejection (TCMR) after transplantation led to the discovery of direct allorecognition.

### Direct T Cell Allorecognition: Molecular Mechanisms of Foreign Cell Communication

**FIGURE 1:**
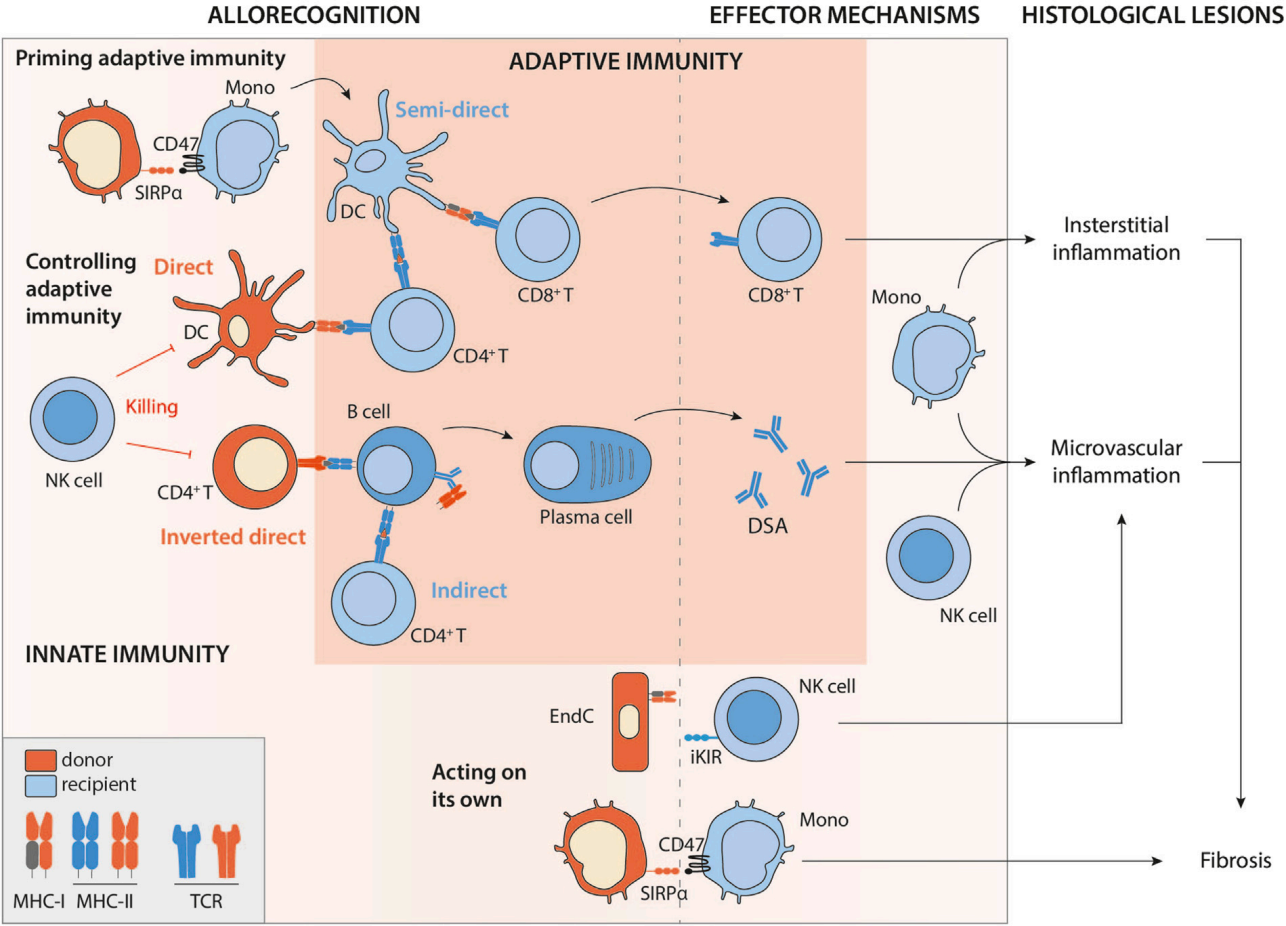
Intricate innate and adaptive allorecognition pathways leading to allograft rejection. Legend: Mono, monocyte; DC, dendritic cell; NK, natural killer; EndC, endothelial cell; MHC, major histocompatibility complex; TCR, T-cell receptor; iKIR, inhibitory killer cell immunoglobulin-like receptor; SIRPα, signal-regulatory protein alpha; DSA, donor specific antibodies.

Direct T cell allorecognition depends on the recognition by recipient T cells of intact allogeneic MHC molecules expressed on graft cells. Left uncontrolled, this phenomenon rapidly leads to TCMR (Figure 1), since 1%–10% of a given individual’s T cell repertoire is capable of recognising intact allogeneic MHC [5, 6].

There are two theories to explain this phenomenon [7–10], restricted to major alloantigens.

**FIGURE 2:**
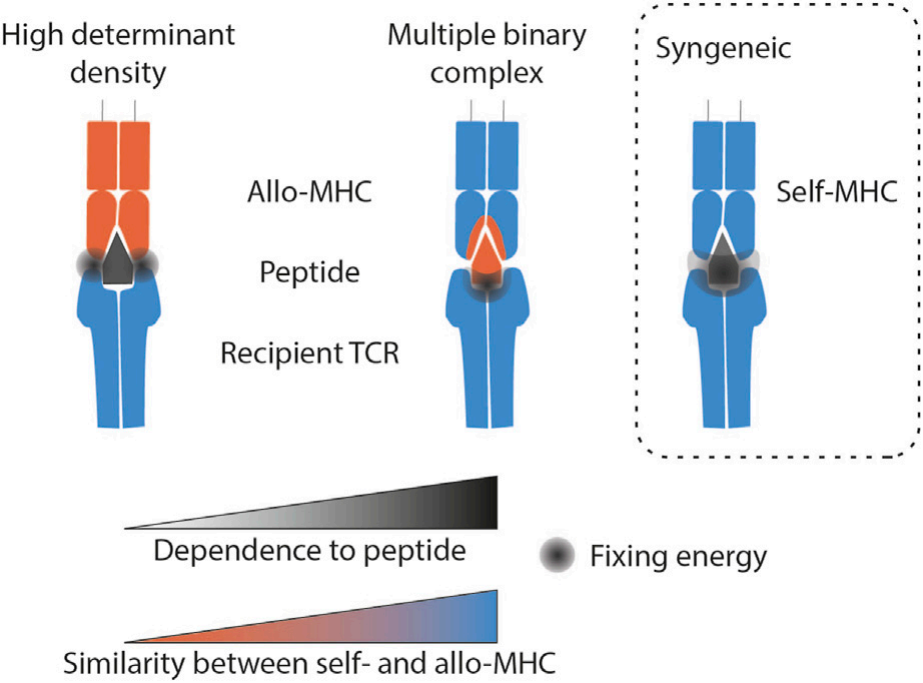
Molecular mechanisms of TCR-mediated direct allorecognition compared with syngeneic recognition. The grey area represents the location where the binding strength of the TCR to the MHC/peptide complex is strongest according to the theory described. Syngeneic recognition implies an interaction force that encompasses the whole complex. The multiple binary complex theory predicts that the ability to recognise endogenous peptides is the main factor determining alloreactivity. Finally, the high determinant density theory proposes that the TCR directly recognises the MHC polymorphism, independently of the peptide antigen. Legend: MHC, Major histocompatibility complex.

The “multiple binary complex” hypothesis (Figure 2) suggests that the principal factor determining the strength of T cell allorecognition is the wide variety of antigenic peptides presented by the donor’s MHC molecules. Because differences in the MHC molecules between donor and recipient are concentrated in the peptide-binding grooves, donor and recipient cells present different peptides derived from the exact same protein. Thus, one donor MHC molecule can potentially represent a myriad of different antigenic complexes depending on the particular peptides bound. The “multiple binary complex” hypothesis was first suggested by the observation that T cell clones that responded to allogeneic antigen presenting cells (APCs) presenting peptides derived from human albumin did not respond to the same APCs presenting peptides from bovine albumin [11]. According to this theory, the ability to recognise endogenous peptides, and not the ability to “directly” interact with allogeneic MHC, is the main factor determining alloreactivity [12–14]. Heterologous immunity is a special case, which nevertheless fits into this theory. During an antiviral response, memory T lymphocytes are generated that recognise a self MHC/viral peptide complex. Molecular mimicry between this complex and donor MHC/peptide can divert the antiviral memory response into an allogeneic response [15, 16]. The multiple binary complex theory also explains the occurrence of TCMR in recipients of HLA-identical transplants. In this case, alloreactivity is not driven by polymorphisms in the binding groove (it is identical in donor and recipient), but rather by the fact that intra-familial HLA-identical donors are not genetically identical like monozygotic twins. While they may share the same HLA genes, the rest of their genomes can differ significantly.

Finally, it shall be kept in mind that the expression of MHC-I-peptide complexes on the cell surface depends on the function of various intracellular assembly factors, such as the transporter associated with antigen presentation (TAP), tapasin, calreticulin, ERp57, TAP-binding protein related (TAPBPR), endoplasmic reticulum aminopeptidases (ERAPs), and proteasomes. It is therefore conceivable that polymorphism in these proteins also contribute to differences in the spectrum of HLA bound peptides (independently of differences in HLA binding groove and polymorphism of minor histocompatibility antigen) [17, 18].

According to the second theory – “the high determinant density theory” - the TCR directly recognises the MHC polymorphism, independently of the peptide antigen (Figure 2). Therefore, because potentially every peptide-MHC complex on the surface of the allogeneic APC is recognised as foreign, a strong overall response is triggered, even though the strength of the interaction between the TCR and individual alloMHC/peptide complexes may be very low [19].

These cognitive studies were mainly conducted in mouse models, or on a few specific HLA molecules, which do not encompass the complexity and extreme variability of HLA in humans. It is reasonable to think that for a particular donor/recipient pairing, the modality of interaction between the recipient TCR and the donor MHC lies within a range between the two theories described above.

### Direct Allorecognition: The Tree That Hides the Forest

**FIGURE 3:**
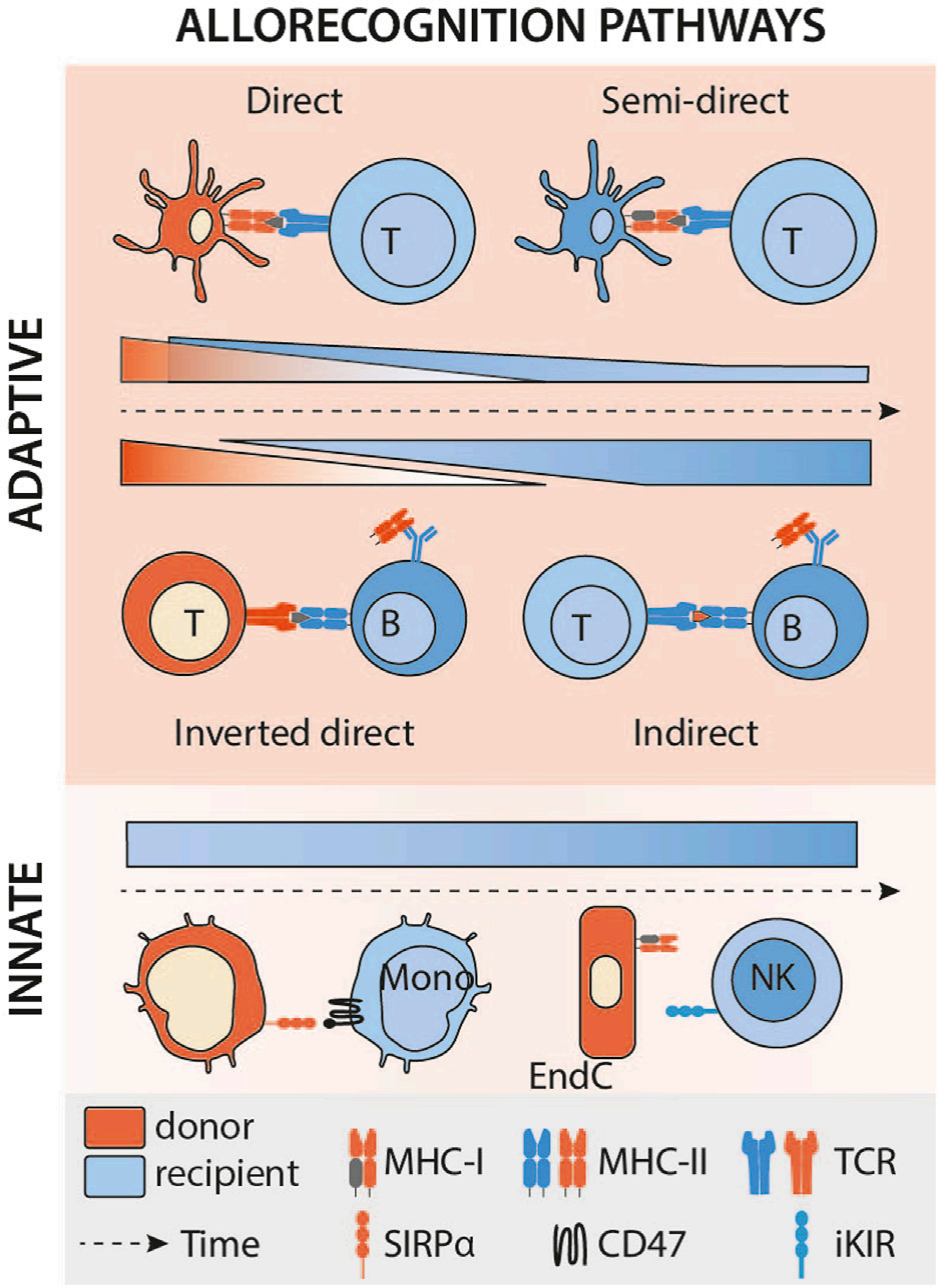
Kinetics of the different allorecognition pathways after transplantation. Legend: Mono, monocyte; DC, dendritic cell; NK, natural killer; EndC, endothelial cell; MHC, major histocompatibility complex; TCR, T-cell receptor; iKIR, inhibitory killer cell immunoglobulin-like receptor; SIRPα, signal-regulatory protein alpha.

In transplantation, it has long been theorised that donor’s leukocytes leave the graft rapidly after transplantation [20]. These passenger leukocytes, in particular the donor’s APCs, reach the recipient’s secondary lymphoid organs (SLOs), and present intact allogeneic MHC molecules on their surface to recipient’s T cells [21]. According to this theory, the incidence of TCMR is directly linked to the presence of donor passenger leukocytes, and it is therefore expected for this incidence to decrease over time as the pool of donor leukocytes fades progressively and cannot be replenished [22] (Figure 3). The fact that vascularised composite allografts (VCA), in which the stock of donor APC can self-renew (i.e; Langerhans cells in the skin), are often targeted by late TCMR, can also be seen as an indirect validation of this theory [23, 24].

The development of an effective cytotoxic response requires the provision of CD4^+^ T cell help to the CD8^+^ T cell [25]. In the context of direct allorecognition, this help can be provided by CD4^+^ T cells with direct specificity [26], presumably because within the recipient’s SLO, a single donor APC can establish a productive three-cell cluster with CD4^+^ and CD8^+^ T cells that directly recognise the MHC class II and class I alloantigens, respectively, on the cell surface (Figure 1).

This allorecognition pathway offers the advantage of a treatment that seems readily available, namely, depletion of passenger leukocytes. Thus, several studies have been able to show, in heart, kidney [27] and liver transplantation [28], that depletion of donor APCs induces prolonged graft survival in the absence of immunosuppressants. However, there is a major bias in these studies: depletion of donor APCs not only eliminates the function they carry, but also the stock of antigens (in particular MHC-II) that they strongly express. Notably, Mandelbrot et al have reported that cardiac allografts from donors whose APCs were unable to deliver costimulatory signals to recipient T cells were still rejected, despite the apparent inability to trigger conventional direct-pathway T cell responses [29].

### A Paradigm Shift: Semi-Direct Recognition Takes the Lead

The limitations discussed above and the observation that TCMR can occur late, at a time when the pool of passenger donor leukocytes is expected to be exhausted, led to reconsideration as to how alloimmune responses with T cells with direct specificity are triggered.

A series of publications from the early 2000s have instead suggested that presentation of MHC alloantigen that has been acquired by the recipient APC and represented intact without processing may be key [30] (Figure 1). These recipient’s APCs could have taken up donor antigens by direct cell-cell contact [31] with either migrating donor leukocytes or graft parenchymal cells (recipient APCs rapidly infiltrate the grafts after transplantation [32]). Alternatively, alloantigen may be acquired from donor-derived exosomes or extracellular vesicles drained from the graft to recipient’s SLOs [33, 34]. It should be noted that donor-derived exosomes cannot activate T cells on their own but must be presented by donor APCs. This capture by recipient’s APCs and the subsequent presentation to T cells only occurs in an inflammatory context [35], possibly explaining the link sometimes observed between infection and rejection, the causality of which has been difficult to establish [36, 37].

The semi-direct pathway has become gradually recognised as an important mechanism leading to TCMR, since studies (almost exclusively on murine models) by independent groups suggest that it elicits much stronger CD8^+^ T cell immunity than interaction of the CD8^+^ T cell with a donor leukocyte, principally because alloantigen from a single donor APC can be presented intact by many-more fold recipient APCs. The semi-direct pathway can still explain the observed kinetics of TCMR after transplantation (Figure 3). Indeed, in animal models of chronic renal graft rejection, cross-dressing, although declining with time, persists for many weeks after transplantation [2]. The initial peak is probably of mixed cause, linked to the donor’s dendritic cells, which remain a major source of alloantigens in the early stages of the transplant, but also to ischaemia-reperfusion lesions, responsible for the immunogenic death of graft cells and the release of extracellular vesicles. Finally, although, as discussed above, the default pathway for provision of CD4^+^ T cell help to alloreactive CD8^+^ T cells is through direct pathway allorecognition of MHC class II alloantigen on the surface of donor APCs, Lee and Auchnicloss’ seminal paper has demonstrated that indirect-pathway CD4^+^ T cells can also provide effective help for generating effector cytotoxic CD8^+^ T cell alloimmunity [38]. Explaining this phenomenon through the immunological paradigms prevalent at publication was not straightforward, because it necessitated formation of a cumbersome four cell cluster, comprising a donor dendritic cell presenting intact MHC class I alloantigen to an alloreactive CD8^+^ T cell, and a recipient dendritic cell presenting processed alloantigen to an indirect-pathway helper CD4^+^ T cell, with moreover no apparent physical linkage between the former two and the latter two cell types. This risks uncontrolled and damaging bystander T cell activation. Semi-direct recognition provides an elegant solution to obviate these concerns, because, assuming a single recipient dendritic cell presents both intact MHC class I alloantigen and self-MHC class II restricted allopeptide for, respectively, direct-pathway CD8^+^ and indirect-pathway CD4^+^ T cell allorecognition, this recreates a 3-cell cluster model (Figure 1), with physical linkage possible between the three [39–41].

## Allorecognition Pathways Leading to Antibody-Mediated Rejection

Although the development of modern immunosuppressive treatments has not completely eradicated TCMR (particularly late rejection and its complications), it has nevertheless improved short-term graft survival. However, the improvement in the long-term graft outcomes has not been as spectacular, as the rates of graft loss beyond the first year have shown more limited progress over different transplant eras [42–46]. This most likely reflects the contribution of humoral alloimmunity to chronic rejection, with the development of donor specific alloantibody (DSA) increasingly emphasised as an important effector of chronic graft damage [47]. This section considers the different allorecognition pathways responsible for DSA production.

### The Critical Role of Indirect Allorecognition Pathway in DSA Generation

Although, as discussed, indirect pathway of allorecognition can provide help for generating cellular cytototoxic alloimmunity, its role in the provision of help to allospecific B cells is also important for determining graft outcomes. Indirect allorecognition is akin to conventional CD4^+^ T cell recognition of model protein antigen, whereby alloantigen is presented as processed peptide held in the binding groove of the recipient’s MHC class II antigen. Essential help for thymo (T) dependent antibody responses is provided by CD4^+^ T cells that similarly recognise complexes of MHC class II and bound peptide antigen, after that antigen has been internalised and processed via the B cell receptor (BCR). Thus, recipient indirect-pathway CD4^+^ T cells provide help for generating alloantibody [48–50] (Figure 1). The scientific literature on T-dependent humoral immunity has expanded prolifically over the last decade, with several recent publications considering how these advances shape our understanding of the B cell alloresponse.

Unlike cellular alloimmunity, whose functional relevance is confined to the response against donor major histocompatibility alloantigens [51], the humoral alloimmune response can be initiated by all alloantigens, both major and minor [52]. Alloreactive B cell responses are targeted generally against intact, conformational antigens [53, 54], which means that alloantigens must be transported from the graft to the SLOs for presentation to B cells. This may be achieved by exodus of donor DCs from the graft [20]. The graft may also release extra-cellular vesicles covered with donor MHC that are then captured by recipient DCs [54] or by subcapsular sinus macrophages in lymph nodes (or their equivalents in the spleen) [55] for presentation to allospecific B cells. In parallel, recipient DCs will acquire alloantigen either within SLOs or the graft itself and present it to indirect-pathway CD4^+^ T cells [34], which then migrate to the border of B cell zone and provide cognate B cell help for triggering initial production of class-switched alloantibody via short-lived extrafollicular foci. It is, however, the subsequent relocation to the B cell follicle and the formation of the Germinal Centre (GC) reaction, with essential help provided by further differentiation of indirect-pathway CD4^+^ T follicular helper cells [49], that likely determines transplant outcome. GC responses are uniquely capable of producing somatically-mutated, high-affinity alloantibody and animal models have confirmed the essential role of the GC response in mediating chronic antibody mediated rejection [56, 57]. The GC reaction also produces robust humoral memory, composed of memory B cells and bone-marrow resident, long-lived-plasma cells, with the latter potentially capable of producing alloantibody for the life of the individual, long after the GC response has dissipated. It is this aspect of the GC reaction that makes treatment of AMR and the desensitisation of patients awaiting transplantation so challenging [58, 59].

### Direct Allorecognition and DSA Generation: The Inverted Direct Pathway

Clinicians’ experience and some studies have reported unusually strong *de novo* DSA responses developing early (within the first week) following lung and intestinal transplantation [60]. This early onset of *de novo* DSA suggests that in addition to the canonical indirect pathways, other mechanisms may be responsible for an alloimmune humoral response. In line with this hypothesis, recent work, published independently by Pettigrew’s and our own team [61, 62], has highlighted a major role for passenger donor CD4^+^ T cells that are contained in vascularised grafts and transferred to the recipient after the transplantation. This passenger CD4^+^ T cell population would be expected to contain a relatively large proportion (1%–10%) of cells with direct allospecificity for the recipient’s MHC class II antigens [what is true for recipient cells is also true for donor-derived cells [5, 6]], and are therefore capable of binding with these antigens on the surface of recipient haematopoietic cells. Thus, during inverted direct allorecognition (Figure 1), initial activation of recipient alloreactive B cells is triggered by binding B cell receptor (BCR) to its target alloantigen (as occurs with encounter with any classical antigen). Internalising and processing of the bound alloantigen results in upregulated expression of surface MHC class II presenting bound peptide alloantigen, but rather than cognate help being provided by indirect-pathway CD4^+^ T cells, these MHC class II complexes are recognised in a peptide-degenerate manner by activated passenger donor CD4^+^ T cells, which in turn deliver a second (costimulatory) signal to the B cell, enabling it to differentiate into a DSA-producing plasma cell [61, 62].

To date, a definitive analysis of how the kinetics of DSA production via the inverted direct pathway compares to conventional indirect-pathway CD4^+^ T cell help has not been performed, and it remains unclear whether the very early onset of alloantibody production (as soon as day 7) is mediated exclusively via the inverted direct pathway (Figure 3). A study of the precursor frequencies of the relative T cell pools mediating each pathway may provide some insight. In the indirect pathway, the frequency of the T cells involved is very low (clonal frequency of around 1/10,000). As a result, time-consuming clonal expansion is necessary to achieve an effective response. In contrast, the recently described pathway involves a very large pool of T cells (1%–10% of the T cells contained in the graft), capable of immediately providing effective assistance to B cells, without amplification. Moreover, the majority of transferred donor CD4^+^ T lymphocytes with direct allospecificity for the recipient would likely exhibit a memory phenotype, because of cross-reactive heterologous immunity [15, 16], one would expect that this would provide robust help for even more rapid production of alloantibody [50, 63]. This also explains the seemingly paradoxical finding that heart grafts from donors sensitised to recipients were rejected more rapidly than grafts from naïve donors [61].

In clinical terms, the incidence of early DSAs is immediately correlated with the richness of the graft in lymphoid cells: renal grafts produce very few early DSAs, whereas lung and intestinal grafts (which contain a mucosa-associated lymphoid tissue) induce a massive wave of early *de novo* DSAs, in 25%–80% of patients [62]. It is also worth noting that B cells activated via inverted direct allorecognition should also be capable of ‘soliciting’ their own help from recipient CD4^+^ T cells [64]. Our recent work has suggested that whereas passenger donor CD4^+^ T cells trigger a rapid humoral response in the recipient, maintenance of this response as a GC reaction was dependent upon secondary differentiation of allopeptide-specific T follicular helper cells of recipient origin [57, 65].

One further possible consequence of inverted direct recognition is that, because the help provided by the donor CD4^+^ T cells is likely to be promiscuous and provided to all B cells in a peptide-degenerate fashion, the limiting factor in antibody production is availability of target antigen to bind the BCR. Consequently, in some murine models, inverted direct recognition was associated with production of class-switched anti-nuclear autoantibody responses [65]. Thus, this phenomenon may be responsible for the various autoantibody responses that have been detailed in human transplant recipients [66].

## Innate Allorecognition

In the same way that improved prevention of TCMR has lifted the veil on the clinical importance of AMR, dissection of the pathophysiological mechanisms of the latter has revealed other holes in the picture: not all allograft rejection depends on the adaptive immune system and evidence is increasing for a direct role of innate immunity in allorecognition and graft injury [67].

### Innate Lymphoid Cells-Mediated Allorecognition: A Double-Edged Sword

NK cells are prototypical of the paradigm shift from adaptive to innate rejection. Long implicated as secondary effectors of the humoral arm through their ability to mediate antibody-dependent cellular cytotoxicity (ADCC), they may have additional and independent role in allorecognition and innate allograft damage.

#### NK Cells Control Direct Recognition-Mediated Pathways

The first proof of the involvement of NK-mediated allorecognition was provided for the benefit of graft survival. In fact, NK cells can control the survival of donor cells responsible for direct antigen presentation (Figure 1). This phenomenon was first demonstrated in mice by transplanting organs whose survival was threatened solely by the cellular arm of the adaptive alloimmune response (because the vascularisation comes from the recipient and cannot be targeted by DSA, namely skin and pancreatic islets [68]). In these models, recipient alloreactive NK cells can destroy donor APCs and thus significantly prolong graft survival [69, 70]. More recently, the evidence has been extended to a lung transplantation model, in a study demonstrating that when the recipient’s NK cells are able to eliminate the donor’s DCs, the alloreactive T cell infiltrate is significantly reduced, and the lungs are less rejected [71]. Similarly, donor NK cells are able to eliminate donor CD4^+^ T cells involved in the inverted direct allorecognition pathway [61]. As a result, the latter cannot interact with the recipient’s alloreactive B cells, and the production of early DSAs is avoided (Figure 1). In all these studies, it is accepted that a MHC-I mismatch activates the recipient’s NK cells [72], because the donor’s MHC class I (H2D^d^) is an activating ligand for the Ly49D NK cell receptor in the recipient mouse.

NK cell alloreactivity is however complex because NKs are constantly integrating numerous activating and inhibiting signals, via various membrane receptors. A lack of inhibition (for example, due to the lack of expression on the surface of a target cell of self-MHC molecules, i.e., “missing-self”) or an excess of activation (for example, expression of a stress ligand by the target cell) can lead to activation of the NK cell. In humans, several layers of complexity make difficult the prediction of NK cell behaviour towards allogeneic targets. First, HLA mismatches between donor and recipient, along with the diversity of receptors on the recipient’s NK cells, are the primary determinants of this alloreactivity. Second, the signals delivered by the target can be influenced not only by the quantity but also by the quality (affinity) of the ligands for NK cell receptors. Third, the ability to activate NK cells also depends on the condition of the target (whether stressed or not). Consequently, predicting the behaviour of NK cells towards a specific allogeneic target is currently impossible. This unpredictability extends to interactions between recipient NK cells and a self-APC that cross-presents alloantigens (i.e., an APC that expresses both self and non-self MHC). Since we cannot determine which activating and inhibitory signals will dominate, the control of the semi-direct pathway by NK cells has not been formally evaluated. Notwithstanding, boosting allogeneic NK innate responses at an opportune moment, i.e., in the first few days following organ transplantation, may prove to be an effective lever for limiting both cell rejection and early DSA production.

#### … But NK Cells can Also Directly Damage the Graft

It is now widely accepted that NK cell-mediated allorecognition can also lead to chronic allograft vascular rejection, independently of DSAs. Indeed, it has been demonstrated in mouse models [73] and then confirmed in clinical studies [73, 74] that the lack of expression of self MHC-I by graft vasculature can activate NK cells and cause chronic vascular damages (Figure 1). Of note, about 30% of patients with microvascular inflammation in absence of DSA have also no missing self to explain the histological lesions [73]. Missing-self is therefore likely not the only molecular mechanism able to trigger NK cell response against a graft endothelium and several situations resulting in either an excess of activating signals or a defect in inhibitory signals (or a mixture of both) involving KIR- or NKG2-family of NK receptors could trigger NK cell response against a graft endothelium (reviewed in [75, 76]).

### Innate Myeloid Cells-Mediated Allorecognition: Last Discovery, First to Initiate Rejection

For the sake of clarity, we have separated in this review the mechanisms of allorecognition dependent on innate immunity from those dependent on adaptive immunity, but this last part brings us back to the very beginning of this review: the priming of alloreactive T cells.

The initiation of a T cell response requires the activation and full differentiation of APCs. According to the danger theory, this activation is triggered by danger-associated molecular patterns (DAMPs), molecules of all kinds that can be released by certain types of cell death (necroptosis for example). However, in the context of transplantation, the danger is probably not sufficient to initiate an alloimmune response. Indeed, recent work in mice has unequivocally demonstrated that i) the mouse immune system (particularly monocytes) can distinguish between self and non-self [77] and that ii) this recognition of non-self by monocytes is necessary for the development of an alloimmune T response [78]. Indeed, in syngeneic transplantation, monocytes that differentiate into DCs are incapable of sustaining a T response despite the DAMPs provided by ischaemia-reperfusion inherent in transplantation. In contrast, in an allogeneic context, monocyte-derived DCs induced by allogeneic cells are perfectly mature, express IL-12 and effectively stimulate T cell proliferation. This mechanism of innate myeloid recognition is independent and acts on its own account, since it does not depend on DAMPs receptors [78]. Indeed, the same authors demonstrated that it depends (at least in part) on the recognition, by the recipient’s monocytic CD47, of signal-regulatory protein (SIRP) α polymorphisms in the donor [79] (Figure 1).

Myeloid allorecognition can also act on its own behalf. Monocytes carry paired Ig-like receptors (PIR) which can recognise allogeneic MHC-I molecules on the surface of donor cells. Monocytes activated in this way can contribute to allograft rejection, and even establish an innate memory response [80]. The establishment of memory via PIRs requires the SIRPα signal described above at the time of monocyte priming [79, 80].

These recent findings have yet to be formally confirmed in humans. Recent studies have characterised an infiltrate of non-classical monocytes expressing both CD47 and leukocyte immunoglobulin-like receptors (LILRs, orthologs of murine PIRs) in rejection biopsies of kidney grafts [81, 82], and suggest that these monocytes may support the CD8^+^ T cytotoxic response [82]. Further studies are needed to confirm the role of innate myeloid allorecognition in human transplantation.

## Conclusion

In conclusion, the mechanisms involved in allorecognition are far more complex than initially understood. The direct allorecognition pathway does not fully account for the persistence and dynamics of TCMR episodes. Recent studies have highlighted the importance of the semi-direct pathway, where recipient APCs acquire and present donor MHC molecules. AMR, which depends on the generation of donor-specific antibodies (DSAs), involves both the indirect and direct allorecognition pathways. The indirect pathway engages recipient APCs to present donor peptides to T cells, which then assist B cells in producing DSAs. In contrast, the recently discovered inverted direct pathway involves donor CD4^+^ T cells within the graft directly driving recipient B cells to rapidly produce DSAs.

Additionally, innate allorecognition mechanisms have emerged, supplementing the understanding traditionally dominated by adaptive immunity. On the one hand, NK cells, typically associated with antibody-dependent cellular cytotoxicity, have been shown to mediate direct antigen recognition and impact graft survival through mechanisms like “missing-self”. This has direct clinical implications, because DSA-independent missing self-induced microvascular inflammation, which was classified as “no rejection” according to Banff 2019 [83], now falls into a subcategory of DSA-negative and C4d-negative microvascular inflammation (Banff 2022, [84]). In addition, these microvascular injuries justify a specific, tailored treatment that does not target DSA but only NK, and which is currently being evaluated in a clinical trial. Still on the subject of NK cells, their involvement in controlling the inverted direct pathway in the clinic has yet to be demonstrated. If this were the case, it would pave the way for treatments to modulate NK alloreactivity according to the time of transplantation, by stimulating it at the start of the graft to prevent the formation of early DSA, and by repressing it thereafter to prevent chronic vascular rejection. On the other hand, the involvement of innate myeloid cells, particularly monocytes, in recognizing non-self and initiating alloimmune responses highlights the variety and complexity of allorecognition. However, evidence in humans is still lacking and requires dedicated studies before monocytes can be considered as a therapeutic target.

This expanded understanding of allorecognition is crucial for developing more effective strategies to manage and prevent graft rejection, ultimately improving the outcomes of organ and tissue transplants. These insights highlight the need for a comprehensive approach in managing transplant rejection, integrating targeted strategies against both adaptive and innate immune mechanisms to improve long-term outcomes.

## References

[1] Reindl-Schwaighofer R Heinzel A Kainz A van Setten J Jelencsics K Hu K Contribution of Non-HLA Incompatibility between Donor and Recipient to Kidney Allograft Survival: Genome-Wide Analysis in a Prospective Cohort. The Lancet (2019) 393(10174):910–7. 10.1016/S0140-6736(18)32473-5 30773281

[2] Hughes AD Zhao D Dai H Abou-Daya KI Tieu R Rammal R Cross-Dressed Dendritic Cells Sustain Effector T Cell Responses in Islet and Kidney Allografts. J Clin Invest (2020) 130(1):287–94. 10.1172/JCI125773 31763998 PMC6934226

[3] Ashby KM Hogquist KA. A Guide to Thymic Selection of T Cells. Nat Rev Immunol (2024) 24(2):103–17. 10.1038/s41577-023-00911-8 37464188

[4] Zinkernagel RM Doherty PC. Restriction of In Vitro T Cell-Mediated Cytotoxicity in Lymphocytic Choriomeningitis within a Syngeneic or Semiallogeneic System. Nature (1974) 248(5450):701–2. 10.1038/248701a0 4133807

[5] Veerapathran A Pidala J Beato F Yu XZ Anasetti C. *Ex vivo* Expansion of Human Tregs Specific for Alloantigens Presented Directly or Indirectly. Blood (2011) 118(20):5671–80. 10.1182/blood-2011-02-337097 21948174 PMC3217366

[6] Suchin EJ Langmuir PB Palmer E Sayegh MH Wells AD Turka LA. Quantifying the Frequency of Alloreactive T Cells *In Vivo*: New Answers to an Old Question. J Immunol (2001) 166(2):973–81. 10.4049/jimmunol.166.2.973 11145675

[7] Game DS Lechler RI. Pathways of Allorecognition: Implications for Transplantation Tolerance. Transpl Immunol (2002) 10(2):101–8. 10.1016/s0966-3274(02)00055-2 12216939

[8] Afzali B Lombardi G Lechler RI. Pathways of Major Histocompatibility Complex Allorecognition. Curr Opin Organ Transpl (2008) 13(4):438–44. 10.1097/MOT.0b013e328309ee31 PMC3815495 18685342

[9] Archbold JK Macdonald WA Miles JJ Brennan RM Kjer-Nielsen L McCluskey J Alloreactivity between Disparate Cognate and Allogeneic pMHC-I Complexes Is the Result of Highly Focused, Peptide-dependent Structural Mimicry. J Biol Chem (2006) 281(45):34324–32. 10.1074/jbc.M606755200 16963442

[10] Boardman DA Jacob J Smyth LA Lombardi G Lechler RI. What Is Direct Allorecognition? Curr Transpl Rep (2016) 3(4):275–83. 10.1007/s40472-016-0115-8 27909647 PMC5107184

[11] Panina-Bordignon P Corradin G Roosnek E Sette A Lanzavecchia A. Recognition by Class II Alloreactive T Cells of Processed Determinants From Human Serum Proteins. Science (1991) 252(5012):1548–50. 10.1126/science.1710827 1710827

[12] Morris GP Ni PP Allen PM. Alloreactivity Is Limited by the Endogenous Peptide Repertoire. Proc Natl Acad Sci U S A (2011) 108(9):3695–700. 10.1073/pnas.1017015108 21321209 PMC3048116

[13] Son ET Faridi P Paul-Heng M Leong ML English K Ramarathinam SH The Self-Peptide Repertoire Plays a Critical Role in Transplant Tolerance Induction. J Clin Invest (2021) 131(21):e146771. 10.1172/JCI146771 34428180 PMC8553557

[14] Abdelsamed HA Lakkis FG. The Role of Self-Peptides in Direct T Cell Allorecognition. J Clin Invest (2021) 131(21):e154096. 10.1172/JCI154096 34720090 PMC8553548

[15] Adams AB Pearson TC Larsen CP. Heterologous Immunity: An Overlooked Barrier to Tolerance. Immunol Rev (2003) 196(1):147–60. 10.1046/j.1600-065x.2003.00082.x 14617203

[16] Adams AB Williams MA Jones TR Shirasugi N Durham MM Kaech SM Heterologous Immunity Provides a Potent Barrier to Transplantation Tolerance. J Clin Invest (2003) 111(12):1887–95. 10.1172/JCI17477 12813024 PMC161424

[17] Powis SJ Deverson EV Coadwell WJ Ciruela A Huskisson NS Smith H Effect of Polymorphism of an MHC-Linked Transporter on the Peptides Assembled in a Class I Molecule. Nature (1992) 357(6375):211–5. 10.1038/357211a0 1350326

[18] Zaitoua AJ Kaur A Raghavan M. Variations in MHC Class I Antigen Presentation and Immunopeptidome Selection Pathways. F1000Research (2020) 9:F1000 Faculty Rev-1177. 10.12688/f1000research.26935.1 PMC7525337 33014341

[19] Villadangos JA Galocha B López de Castro JA. Unusual Topology of an HLA-B27 Allospecific T Cell Epitope Lacking Peptide Specificity. J Immunol Baltim Md (1994) 152(5):2317–23. 10.4049/jimmunol.152.5.2317 7510742

[20] Celli S Albert ML Bousso P. Visualizing the Innate and Adaptive Immune Responses Underlying Allograft Rejection by Two-Photon Microscopy. Nat Med (2011) 17(6):744–9. 10.1038/nm.2376 21572426

[21] Larsen CP Morris PJ Austyn JM. Migration of Dendritic Leukocytes from Cardiac Allografts into Host Spleens. A Novel Pathway for Initiation of Rejection. J Exp Med (1990) 171(1):307–14. 10.1084/jem.171.1.307 2404081 PMC2187651

[22] Halloran PF Chang J Famulski K Hidalgo LG Salazar IDR Merino Lopez M Disappearance of T Cell-Mediated Rejection Despite Continued Antibody-Mediated Rejection in Late Kidney Transplant Recipients. J Am Soc Nephrol JASN (2015) 26(7):1711–20. 10.1681/ASN.2014060588 25377077 PMC4483591

[23] Kanitakis J Morelon E Petruzzo P Badet L Dubernard JM. Self-renewal Capacity of Human Epidermal Langerhans Cells: Observations Made on a Composite Tissue Allograft. Exp Dermatol (2011) 20(2):145–6. 10.1111/j.1600-0625.2010.01146.x 20707812

[24] Thaunat O Badet L Dubois V Kanitakis J Petruzzo P Morelon E. Immunopathology of Rejection: Do the Rules of Solid Organ Apply to Vascularized Composite Allotransplantation? Curr Opin Organ Transpl (2015) 20(6):596–601. 10.1097/MOT.0000000000000242 26536419

[25] Bevan MJ. Helping the CD8(+) T-Cell Response. Nat Rev Immunol (2004) 4(8):595–602. 10.1038/nri1413 15286726

[26] Taylor AL Negus SL Negus M Bolton EM Bradley JA Pettigrew GJ. Pathways of Helper CD4 T Cell Allorecognition in Generating Alloantibody and CD8 T Cell Alloimmunity. Transplantation (2007) 83(7):931–7. 10.1097/01.tp.0000257960.07783.e3 17460565

[27] Brown K Nowocin AK Meader L Edwards LA Smith RA Wong W. Immunotoxin against a Donor MHC Class II Molecule Induces Indefinite Survival of Murine Kidney Allografts. Am J Transpl (2016) 16(4):1129–38. 10.1111/ajt.13584 PMC4988511 26799449

[28] Ueta H Xu XD Yu B Kitazawa Y Yu E Hara Y Suppression of Liver Transplant Rejection by Anti-Donor MHC Antibodies via Depletion of Donor Immunogenic Dendritic Cells. Int Immunol (2021) 33(5):261–72. 10.1093/intimm/dxaa076 33258927 PMC8060989

[29] Mandelbrot DA Furukawa Y McAdam AJ Alexander SI Libby P Mitchell RN Expression of B7 Molecules in Recipient, Not Donor, Mice Determines the Survival of Cardiac Allografts. J Immunol Baltim Md (1999) 163(7):3753–7. 10.4049/jimmunol.163.7.3753 10490971

[30] Herrera OB Golshayan D Tibbott R Ochoa FS James MJ Marelli-Berg FM A Novel Pathway of Alloantigen Presentation by Dendritic Cells. J Immunol (2004) 173(8):4828–37. 10.4049/jimmunol.173.8.4828 15470023

[31] Russo V Zhou D Sartirana C Rovere P Villa A Rossini S Acquisition of Intact Allogeneic Human Leukocyte Antigen Molecules by Human Dendritic Cells. Blood (2000) 95(11):3473–7. 10.1182/blood.v95.11.3473.011k06_3473_3477 10828031

[32] Zhuang Q Liu Q Divito SJ Zeng Q Yatim KM Hughes AD Graft-infiltrating Host Dendritic Cells Play a Key Role in Organ Transplant Rejection. Nat Commun (2016) 7(1):12623. 10.1038/ncomms12623 27554168 PMC4999515

[33] Marino J Babiker-Mohamed MH Crosby-Bertorini P Paster JT LeGuern C Germana S Donor Exosomes Rather Than Passenger Leukocytes Initiate Alloreactive T Cell Responses after Transplantation. Sci Immunol (2016) 1(1):aaf8759. 10.1126/sciimmunol.aaf8759 27942611 PMC5142759

[34] Liu Q Rojas-Canales DM Divito SJ Shufesky WJ Stolz DB Erdos G Donor Dendritic Cell-Derived Exosomes Promote Allograft-Targeting Immune Response. J Clin Invest (2016) 126(8):2805–20. 10.1172/JCI84577 27348586 PMC4966303

[35] Prunevieille A Babiker-Mohamed MH Aslami C Gonzalez-Nolasco B Mooney N Benichou G. T Cell Antigenicity and Immunogenicity of Allogeneic Exosomes. Am J Transpl (2021) 21(7):2583–9. 10.1111/ajt.16591 PMC10601455 33794063

[36] Wang T Ahmed EB Chen L Xu J Tao J Wang CR Infection with the Intracellular Bacterium, Listeria Monocytogenes, Overrides Established Tolerance in a Mouse Cardiac Allograft Model. Am J Transpl (2010) 10(7):1524–33. 10.1111/j.1600-6143.2010.03066.x PMC4060596 20642679

[37] Cainelli F Vento S. Infections and Solid Organ Transplant Rejection: A Cause-And-Effect Relationship? Lancet Infect Dis (2002) 2(9):539–49. 10.1016/s1473-3099(02)00370-5 12206970

[38] Lee RS Grusby MJ Glimcher LH Winn HJ Auchincloss H Jr. Indirect Recognition by Helper Cells Can Induce Donor-Specific Cytotoxic T Lymphocytes In Vivo. J Exp Med (1994) 179(3):865–72. 10.1084/jem.179.3.865 8113680 PMC2191395

[39] Brown K Sacks SH Wong W. Coexpression of Donor Peptide/Recipient MHC Complex and Intact Donor MHC: Evidence for a Link between the Direct and Indirect Pathways. Am J Transpl (2011) 11(4):826–31. 10.1111/j.1600-6143.2011.03437.x 21401861

[40] Sivaganesh S Harper SJ Conlon TM Callaghan CJ Saeb-Parsy K Negus MC Copresentation of Intact and Processed MHC Alloantigen by Recipient Dendritic Cells Enables Delivery of Linked Help to Alloreactive CD8 T Cells by Indirect-Pathway CD4 T Cells. J Immunol Baltim Md (2013) 190(11):5829–38. 10.4049/jimmunol.1300458 PMC3736307 23630361

[41] Harper SJF Ali JM Wlodek E Negus MC Harper IG Chhabra M CD8 T-Cell Recognition of Acquired Alloantigen Promotes Acute Allograft Rejection. Proc Natl Acad Sci (2015) 112(41):12788–93. 10.1073/pnas.1513533112 26420874 PMC4611606

[42] Lamb KE Lodhi S Meier-Kriesche HU. Long-Term Renal Allograft Survival in the United States: A Critical Reappraisal. Am J Transpl (2011) 11(3):450–62. 10.1111/j.1600-6143.2010.03283.x 20973913

[43] Coemans M Callemeyn J Naesens M. Long-Term Survival after Kidney Transplantation. N Engl J Med (2022) 386(5):497–8. 10.1056/NEJMc2115207 35108480

[44] Poggio ED Augustine JJ Arrigain S Brennan DC Schold JD. Long-term Kidney Transplant Graft Survival-Making Progress When Most Needed. Am J Transpl Off J Am Soc Transpl Am Soc Transpl Surg (2021) 21(8):2824–32. 10.1111/ajt.16463 33346917

[45] Sexton DJ O’Kelly P Williams Y Plant WD Keogan M Khalib K Progressive Improvement in Short-Medium- and Long-Term Graft Survival in Kidney Transplantation Patients in Ireland - a Retrospective Study. Transpl Int Off J Eur Soc Organ Transpl (2019) 32(9):974–84. 10.1111/tri.13470 31209932

[46] Coemans M Süsal C Döhler B Anglicheau D Giral M Bestard O Analyses of the Short- and Long-Term Graft Survival After Kidney Transplantation in Europe between 1986 and 2015. Kidney Int (2018) 94(5):964–73. 10.1016/j.kint.2018.05.018 30049474

[47] Terasaki PI. Humoral Theory of Transplantation. Am J Transpl (2003) 3(6):665–73. 10.1034/j.1600-6143.2003.00135.x 12780557

[48] Steele DJ Laufer TM Smiley ST Ando Y Grusby MJ Glimcher LH Two Levels of Help for B Cell Alloantibody Production. J Exp Med (1996) 183(2):699–703. 10.1084/jem.183.2.699 8627185 PMC2192460

[49] Conlon TM Saeb-Parsy K Cole JL Motallebzadeh R Qureshi MS Rehakova S Germinal Center Alloantibody Responses Are Mediated Exclusively by Indirect-Pathway CD4 T Follicular Helper Cells. J Immunol (2012) 188(6):2643–52. 10.4049/jimmunol.1102830 22323543 PMC3378630

[50] Conlon TM Cole JL Motallebzadeh R Harper I Callaghan CJ Bolton EM Unlinked Memory Helper Responses Promote Long-Lasting Humoral Alloimmunity. J Immunol (2012) 189(12):5703–12. 10.4049/jimmunol.1202257 23162131 PMC3788591

[51] Valujskikh A Zhang Q Heeger PS. CD8 T Cells Specific for a Donor-Derived, Self-Restricted Transplant Antigen Are Nonpathogenic Bystanders After Vascularized Heart Transplantation in Mice1. J Immunol (2006) 176(4):2190–6. 10.4049/jimmunol.176.4.2190 16455975

[52] Reindl-Schwaighofer R Heinzel A Signorini L Thaunat O Oberbauer R. Mechanisms Underlying Human Genetic Diversity: Consequence for Antigraft Antibody Responses. Transpl Int Off J Eur Soc Organ Transpl (2018) 31(3):239–50. 10.1111/tri.13059 28865128

[53] Pettigrew GJ Lovegrove E Bradley JA Maclean J Bolton EM. Indirect T Cell Allorecognition and Alloantibody-Mediated Rejection of MHC Class I-Disparate Heart Grafts. J Immunol Baltim Md (1998) 161(3):1292–8. 10.4049/jimmunol.161.3.1292 9686590

[54] Curry AJ Pettigrew GJ Negus MC Easterfield AJ Young JL Bolton EM Dendritic Cells Internalise and Re-present Conformationally Intact Soluble MHC Class I Alloantigen for Generation of Alloantibody. Eur J Immunol (2007) 37(3):696–705. 10.1002/eji.200636543 17266175

[55] Zeng F Chen Z Chen R Shufesky WJ Bandyopadhyay M Camirand G Graft-Derived Extracellular Vesicles Transported Across Subcapsular Sinus Macrophages Elicit B Cell Alloimmunity after Transplantation. Sci Transl Med (2021) 13(585):eabb0122. 10.1126/scitranslmed.abb0122 33731430 PMC8939235

[56] Chhabra M Alsughayyir J Qureshi MS Mallik M Ali JM Gamper I Germinal Center Alloantibody Responses Mediate Progression of Chronic Allograft Injury. Front Immunol (2018) 9:3038. 10.3389/fimmu.2018.03038 30728823 PMC6351502

[57] Qureshi MS Alsughayyir J Chhabra M Ali JM Goddard MJ Devine CA Germinal Center Humoral Autoimmunity Independently Mediates Progression of Allograft Vasculopathy. J Autoimmun (2019) 98:44–58. 10.1016/j.jaut.2018.11.006 30528910

[58] Auner S Hillebrand C Boehm PM Boecker J Koren D Schwarz S Impact of Transient and Persistent Donor-specific Antibodies in Lung Transplantation. Transpl Int Off J Eur Soc Organ Transpl (2024) 37:12774. 10.3389/ti.2024.12774 PMC11110840 38779355

[59] Rostaing LPE Böhmig GA Gibbons B Taqi MM. Post-transplant Surveillance and Management of Chronic Active Antibody-Mediated Rejection in Renal Transplant Patients in Europe. Transpl Int Off J Eur Soc Organ Transpl (2023) 36:11381. 10.3389/ti.2023.11381 PMC10389272 37529383

[60] Ius F Sommer W Tudorache I Kühn C Avsar M Siemeni T Early Donor-Specific Antibodies in Lung Transplantation: Risk Factors and Impact on Survival. J Heart Lung Transpl (2014) 33(12):1255–63. 10.1016/j.healun.2014.06.015 25070908

[61] Harper IG Ali JM Harper SJF Wlodek E Alsughayyir J Negus MC Augmentation of Recipient Adaptive Alloimmunity by Donor Passenger Lymphocytes Within the Transplant. Cell Rep (2016) 15(6):1214–27. 10.1016/j.celrep.2016.04.009 27134179 PMC4870521

[62] Charmetant X Chen CC Hamada S Goncalves D Saison C Rabeyrin M Inverted Direct Allorecognition Triggers Early Donor-specific Antibody Responses After Transplantation. Sci Transl Med (2022) 14(663):eabg1046. 10.1126/scitranslmed.abg1046 36130013

[63] Lovegrove E Pettigrew GJ Bolton EM Bradley JA. Epitope Mapping of the Indirect T Cell Response to Allogeneic Class I MHC: Sequences Shared by Donor and Recipient MHC May Prime T Cells That Provide Help for Alloantibody Production1. J Immunol (2001) 167(8):4338–44. 10.4049/jimmunol.167.8.4338 11591757

[64] Stockinger B Zal T Zal A Gray D. B Cells Solicit Their Own Help From T Cells. J Exp Med (1996) 183(3):891–9. 10.1084/jem.183.3.891 8642293 PMC2192359

[65] Ali JM Negus MC Conlon TM Harper IG Qureshi MS Motallebzadeh R Diversity of the CD4 T Cell Alloresponse: The Short and the Long of it. Cell Rep (2016) 14(5):1232–45. 10.1016/j.celrep.2015.12.099 26804905 PMC5405053

[66] Siu JHY Motallebzadeh R Pettigrew GJ. Humoral Autoimmunity After Solid Organ Transplantation: Germinal Ideas May Not Be Natural. Cell Immunol (2020) 354:104131. 10.1016/j.cellimm.2020.104131 32563029

[67] Uehara H Minami K Quante M Nian Y Heinbokel T Azuma H Recall Features and Allorecognition in Innate Immunity. Transpl Int Off J Eur Soc Organ Transpl (2018) 31(1):6–13. 10.1111/tri.13073 PMC7781186 28926127

[68] Chen CC Pouliquen E Broisat A Andreata F Racapé M Bruneval P Endothelial Chimerism and Vascular Sequestration Protect Pancreatic Islet Grafts From Antibody-Mediated Rejection. J Clin Invest (2018) 128(1):219–32. 10.1172/JCI93542 29202467 PMC5749508

[69] Beilke JN Kuhl NR Kaer LV Gill RG. NK Cells Promote Islet Allograft Tolerance via a Perforin-Dependent Mechanism. Nat Med (2005) 11(10):1059–65. 10.1038/nm1296 16155578

[70] Yu G Xu X Vu MD Kilpatrick ED Li XC. NK Cells Promote Transplant Tolerance by Killing Donor Antigen-Presenting Cells. J Exp Med (2006) 203(8):1851–8. 10.1084/jem.20060603 16864660 PMC2118385

[71] Jungraithmayr W Codarri L Bouchaud G Krieg C Boyman O Gyülvészi G Cytokine Complex–Expanded Natural Killer Cells Improve Allogeneic Lung Transplant Function via Depletion of Donor Dendritic Cells. Am J Respir Crit Care Med (2013) 187(12):1349–59. 10.1164/rccm.201209-1749OC 23590269 PMC3734612

[72] Laffont S Seillet C Ortaldo J Coudert JD Guéry JC. Natural Killer Cells Recruited into Lymph Nodes Inhibit Alloreactive T-Cell Activation Through Perforin-Mediated Killing of Donor Allogeneic Dendritic Cells. Blood (2008) 112(3):661–71. 10.1182/blood-2007-10-120089 18505782

[73] Koenig A Chen CC Marçais A Barba T Mathias V Sicard A Missing Self Triggers NK Cell-Mediated Chronic Vascular Rejection of Solid Organ Transplants. Nat Commun (2019) 10(1):5350. 10.1038/s41467-019-13113-5 31767837 PMC6877588

[74] Callemeyn J Senev A Coemans M Lerut E Sprangers B Kuypers D Missing Self-Induced Microvascular Rejection of Kidney Allografts: A Population-Based Study. J Am Soc Nephrol JASN (2021) 32(8):2070–82. 10.1681/ASN.2020111558 34301794 PMC8455279

[75] Hamada S Dubois V Koenig A Thaunat O. Allograft Recognition by Recipient’s Natural Killer Cells: Molecular Mechanisms and Role in Transplant Rejection. HLA (2021) 98(3):191–9. 10.1111/tan.14332 34050618

[76] Charmetant X Bachelet T Déchanet-Merville J Walzer T Thaunat O. Innate (And Innate-like) Lymphoid Cells: Emerging Immune Subsets With Multiple Roles along Transplant Life. Transplantation (2021) 105(12):e322–e336. 10.1097/TP.0000000000003782 33859152

[77] Zecher D van Rooijen N Rothstein DM Shlomchik WD Lakkis FG. An Innate Response to Allogeneic Nonself Mediated by Monocytes1. J Immunol (2009) 183(12):7810–6. 10.4049/jimmunol.0902194 19923456

[78] Oberbarnscheidt MH Zeng Q Li Q Dai H Williams AL Shlomchik WD Non-self Recognition by Monocytes Initiates Allograft Rejection. J Clin Invest (2014) 124(8):3579–89. 10.1172/JCI74370 24983319 PMC4109551

[79] Dai H Friday AJ Abou-Daya KI Williams AL Mortin-Toth S Nicotra ML Donor SIRPα Polymorphism Modulates the Innate Immune Response to Allogeneic Grafts. Sci Immunol (2017) 2(12):eaam6202. 10.1126/sciimmunol.aam6202 28783664 PMC5653256

[80] Dai H Lan P Zhao D Abou-Daya K Liu W Chen W PIRs Mediate Innate Myeloid Cell Memory to Nonself MHC Molecules. Science (2020) 368(6495):1122–7. 10.1126/science.aax4040 32381589 PMC7379379

[81] Lamarthée B Genet C Cattin F Danger R Giral M Brouard S Single-cell Mapping of Leukocyte Immunoglobulin-like Receptors in Kidney Transplant Rejection. Front Transpl (2022) 1:952785. 10.3389/frtra.2022.952785 PMC11235271 38994376

[82] Lamarthée B Callemeyn J Van Herck Y Antoranz A Anglicheau D Boada P Transcriptional and Spatial Profiling of the Kidney Allograft Unravels a Central Role for FcyRIII+ Innate Immune Cells in Rejection. Nat Commun (2023) 14(1):4359. 10.1038/s41467-023-39859-7 37468466 PMC10356785

[83] Loupy A Haas M Roufosse C Naesens M Adam B Afrouzian M The Banff 2019 Kidney Meeting Report (I): Updates on and Clarification of Criteria for T Cell– and Antibody‐mediated Rejection. Am J Transpl (2020) 20(9):2318–31. 10.1111/ajt.15898 PMC7496245 32463180

[84] Naesens M Roufosse C Haas M Lefaucheur C Mannon RB Adam BA The Banff 2022 Kidney Meeting Report: Reappraisal of Microvascular Inflammation and the Role of Biopsy-Based Transcript Diagnostics. Am J Transpl (2024) 24(3):338–49. 10.1016/j.ajt.2023.10.016 38032300

